# Adiposity and Muscularity Evaluation Using New Objective Morphological Methods Available in Clinical Veterinary Practice: Feline Body Mass Index and Ultrasonography †

**DOI:** 10.3390/ani16040528

**Published:** 2026-02-07

**Authors:** Eiji Iwazaki, Akihiro Mori

**Affiliations:** 1 Department of Research Center No. 2 Section, Nippon Pet Food Co., Ltd., Tokyo 140-0002, Japan; 2 School of Veterinary Nursing and Technology, Faculty of Veterinary Science, Nippon Veterinary and Life Science University, Tokyo 180-0023, Japan; 3 Department of Animal Sciences, University of Illinois, Urbana, IL 61801, USA

**Keywords:** body composition, body mass index, ultrasonography, zoometry measurement, adiposity, muscularity, obesity, sarcopenia, frailty

## Abstract

In this narrative review, we provide an overview of morphology-based body composition measurement techniques and introduce evaluation methods relevant to veterinary practice. We specifically focus on previous work on the feline body mass index and body composition assessment methods using ultrasonography, creating new figures and tables from previously reported data. Based on these modalities, we introduce an objective, easy-to-use method for estimating obesity and assessing body composition. We highlight that adiposity and muscularity evaluation via the feline body mass index and ultrasonography appropriately reflects the total fat mass, fat percentage, and lean body mass assessed using dual-energy X-ray absorptiometry. The proposed method objectively assesses obesity and can be easily and stably used in clinical veterinary practice.

## Simple Summary

We introduce a new method for assessing feline nutritional status that is available in general veterinary practices. We demonstrate that the feline body mass index and the ultrasound evaluation of body fat and muscle mass reflect the gold standard for the assessment of body composition. Adopting this standard in veterinary practice will ensure a high-precision assessment of body fat mass and percentage during health checks or while setting weight reduction targets. We further review nutritional assessment methods used in routine veterinary practice and research, and introduce the latest feline nutritional assessment techniques.

## 1. Introduction

Daily health monitoring at home and veterinary care are important for assessing animal health. The American Animal Hospital Association’s “Nutritional Assessment Guidelines for Dogs and Cats” designate nutritional assessment as the “fifth vital sign” after body temperature, pulse rate, respiratory rate, and blood pressure, and incorporates nutritional screening assessments into regular health screenings [1]. These assessments are also applied by the World Small Animal Veterinary Association (WSAVA), which recommends them as part of a standard physical assessment [2]. Based on these guidelines, screening assessments of adiposity and muscularity focusing on visual inspection and palpation are routinely conducted alongside interviews in general veterinary practice.

Since 2000, 20–60% of cats have been diagnosed as obese or overweight, making obesity a major problem worldwide [3,4,5,6]. Recent research evaluating body condition scores from approximately 1,341,118 cats in electronic medical records at Banfield Pet Hospital in the United States (2020–2023) found that approximately 45–65% of >young adult cats were obese or overweight [7]. Obesity is associated with ectopic fat accumulation and increased concentrations of triglycerides, cholesterol, and non-esterified fatty acids (NEFAs) [8,9,10,11]. Consequently, it induces lipid toxicity in specific tissues, resulting in increased insulin secretion and impaired pancreatic beta cell function, thereby contributing to the onset of severe metabolic syndromes, such as diabetes [8,12,13,14]. In recent years, a relationship between obesity and gut microbiota has been suggested. Yeon et al. demonstrated that the abundance of Bifidobacteriaceae, Corynebacteriaceae, and Veillonellaceae in the gut microbiota of obese cats was positively correlated with their body mass index (BMI) [15].

In contrast, while epidemiological findings on frailty and sarcopenia in cats are limited, a study examining 210 cats aged 11–20 years at a feline specialty hospital found that approximately 51 cats (42%) were frail [16]. Muscle mass in cats decreases with age, and the risk of rapid muscle loss has been suggested to increase particularly in cats aged 10.5 years or older [17].

Furthermore, weight loss treatment often involves a loss of lean body mass (LBM) along with fat reduction in cats [18,19], requiring accurate LBM monitoring techniques.

Obesity and frailty are gaining increased attention, and the value of health screening in veterinary practice is increasingly being recognized. Although body weight is the simplest measure of nutritional status, it cannot distinguish between alterations in body fat, muscle mass, and bone mass.

The Body Condition Score (BCS) and Muscle Mass Score have been recognized by the WSAVA as standards for evaluating adiposity and muscularity [2]. However, these scores are based on subjective assessments via visual inspection and palpation [20,21]. Therefore, they vary significantly depending on the operator’s skill level, resulting in low reproducibility. Thus, an objective technique for the accurate and simultaneous evaluation of adiposity and muscularity is yet to be developed.

The BMI in humans is based on body height and thus independent of fat accumulation. The BMI shows strong correlations with body fat percentage [22,23], abdominal fat accumulation (waist circumference) [24], and hypertension [25], along with the incidence of abnormalities in blood concentrations of energy metabolism products (hyperglycemia, hypertriglyceridemia, hypercholesterolemia, and low/high-density lipoprotein cholesterol) [26]. Moreover, waist circumference is a recognized indicator of obesity, as it reflects abdominal ectopic fat accumulation and is useful for tracking changes within individuals [27].

Similar to human obesity indicators, efforts have been made to develop morphological tools to assess obesity and fat accumulation in cats. However, owing to their limited accuracy and insufficient data, these methods have not been widely adopted in veterinary practice [28,29,30,31,32,33]. Consequently, objective, user-friendly methods for cat owners and more accurate body composition assessment methods for clinical use need to be developed.

In this study, we provide an overview of health diagnostic criteria for cats, with a specific focus on morphology-based techniques to assess body composition, and introduce objective, easy-to-use methods for estimating obesity and assessing body composition.

## 2. Previous Approaches

### 2.1. Simple Evaluation Technique for Adiposity

#### 2.1.1. Body Condition Score

The Body Condition Score (BCS) is a diagnostic criterion for obesity based on visual inspection and palpation. The visibility of the chest and spine, presence of an abdominal waist, and palpation of abdominal fat accumulation were assessed. The WSAVA guidelines are based on the 9-point scale developed by Laflamme [20]. This technique is cost-effective and easy to perform by veterinary professionals and pet owners. However, it is a subjective method, and the results may differ depending on the operator’s skill and experience, making it challenging to consistently achieve reliable diagnoses. Several studies have reported that pet owners tend to underestimate obesity levels more than veterinarians [34,35]. Studies measuring inter-evaluator bias in the BCS found relatively low bias, with a complete agreement rate of approximately 50% [36]. While high accuracy is maintained for extreme comparisons, such as ideal weight versus obesity, a 15.5% risk of classification error was demonstrated for comparisons between ideal weight and overweight, which represent relatively close obesity categories.

Thus, efforts have been made to establish an objective method to evaluate obesity based on skeletal zoometric measurements.

#### 2.1.2. Body Mass Index

In humans, the BMI [body weight/(height)^2^ (kg/m^2^)], established by Matsuzawa et al. [37], is commonly adopted. The BMI has also been incorporated into the diabetes screening guidelines by the World Health Organization (WHO). Additionally, the Ministry of Health, Labour, and Welfare of Japan adopted BMI in the health examination implementation standards established under the Labor Safety and Health Act. BMI is strongly associated with disease incidence, and ideal BMI values have been proposed. The BMI is also highly correlated with body fat percentage, abdominal fat accumulation (waist circumference), and hypertension [22,23,24,38]. Additionally, dyslipidemia prevalence (triglycerides/high- and low-density lipoprotein) increases with increasing BMI [39]. A BMI of 22 is considered appropriate based on disease prevalence [37]. The WHO defines a BMI exceeding 25 as overweight and a BMI exceeding 30 as obese, and classifies obesity into three categories: Class I (30–35), Class II (35–40), and Class III (40 or higher) [40].

Therefore, height, which is based on external skeletal length, is less affected by weight fluctuations and can be used as an indicator of basic body size. Based on the human BMI, several reports on the BMI of cats based on skeletal length have been published.

Nelson et al. [33] were the first to report a cat BMI based on external measurements. The calculation formula is as follows [33]:

(1) $$\mathrm{BMI}\;=\mathrm{body}\;\mathrm{weight}\;\left(\mathrm{kg}\right)/height(m)$$

However, the relevance of blood metabolites, accumulated fat, and metabolic syndrome has not been considered, and the validity of the cat BMI has not been confirmed. Moreover, the measurement points for body length and height (including the joints) are unclear, and because cats lack a consistent upright posture like dogs, obtaining stable measurements in a standing position is challenging. In addition, data on the reproducibility or error of height measurements, such as those introduced by variability among operators, have not been reported. Therefore, the application of this method is challenging, and it has not yet been clinically implemented or routinely used.

#### 2.1.3. Feline Body Mass Index

The feline body mass index (FBMI), developed by Hawthorne and Butterwick [30], is used in some veterinary clinics. The authors developed the FBMI as an indirect method for calculating the body fat percentage using external measurements based on skeletal length. In particular, they developed an FBMI table using chest circumference and leg index measurements (LIM: from the middle of the patella to the dorsal tip of the calcaneal process) [30]. This technique involves measuring the ninth rib circumference (chest circumference) and LIM using a standard measuring tape and indirectly estimating the body fat percentage based on these values. The formula for calculating the body fat percentage is described below (Equation (2)). This is considered an objective method for evaluating obesity. However, this study has several limitations. For instance, the calculation formula is complex, and the position of the ninth rib cannot be determined in cases of severe obesity, making it challenging to accurately measure chest circumference. Additionally, cats, being carnivorous animals, exhibit higher mobility than other animals, and the rib cage repeatedly expands and contracts upon breathing [41], making it challenging to obtain stable measurements. Thus, a certain experience is necessary to obtain accurate measurements. However, owing to the complexity of the calculation formula, it has rarely been used in clinical practice.

Similar to the cat BMI introduced above, data on the reproducibility of the FBMI method have not yet been reported.

When considering the BMI of cats, the effects of body composition, such as fat accumulation and muscle mass, are crucial. Recent findings link ectopic fat accumulation in organs and muscles to metabolic syndrome, which has increased interest in body composition assessment. The following section introduces methods for evaluating body fat accumulation.

### 2.2. Evaluation Methods for Fat Accumulation

Methods for evaluating body composition based on morphometric measurements in mammals, including humans, have been investigated for several years. Most of these methods were designed to assess subcutaneous fat accumulation.

Skinfold calipers have been used since the 1950s, when body composition measurement gained importance [42]. Skinfold calipers measure skin thickness by sandwiching it between two calipers. It was once the most commonly used method for evaluating fat accumulation in humans.

#### 2.2.1. Impedance Method

Body composition in humans is commonly determined using bioelectrical impedance analysis. This method leverages the characteristics of fat tissue, which has a lower electrical conductivity and fewer conduction pathways than other tissues. Moreover, it is non-invasive and combines safety and convenience. The assessment of body fat percentage using this method in cats has been reported by Stanton et al. [43]. The following formulas for calculating body fat percentage and fat mass in cats have been proposed:

(2) $$Percentage\;body\;fat=-0.02(L^{2}/BW)-4.12RF+1.48PC-1.16CTC+92.93$$

(3) $$Fat\;mass\;(kg)=0.04PC-0.0004(L^{2}/BW)-0.08RF+1.11$$

where: BW, body weight; L, body length; RF, right forelimb length; PC, pelvic circumference; CTC, cranial thoracic circumference.

However, considering that Stanton et al. [43] used euthanized cats of normal weight, whether obesity or cachexia can be accurately evaluated in living cats has not yet been verified. Additionally, this method is impractical because it requires electrode insertion via invasive needle puncture. This level of invasiveness is disproportionate to the information obtained, making it difficult for owners to accept in the context of health screening.

In dogs, a non-invasive bioelectrical impedance-based body fat meter has been commercialized (Health Lab, Kao, Tokyo, Japan). However, cats’ skin has a greater range of motion owing to their carnivorous defensive characteristics, making it challenging to maintain stable contact with the electrode surface. Thus, bioelectrical impedance-based body fat monitoring in cats has not yet been commercialized.

#### 2.2.2. Fat Accumulation via the FBMI

FBMI charts are based on the following body fat percentage calculation:

(4) $$Body\;fat\;percentage\;(\%)=\frac{\left(\frac{rib\;cage}{0.7067}-\mathrm{LIM}\right)}{0.9156}-\mathrm{LIM}$$

Additionally, German and Martin [41] introduced the following equation:

(5) $$Body\;fat\;percentage\;\left(\%\right)=1.54\;rib\;cage-1.58\;\mathrm{LIM}-8.67$$

These methods are indirect but objectively evaluate a cat’s body fat percentage. However, cats demonstrate a greater range of motion than other animals, and respiratory movements considerably affect their chest circumference, making it challenging to reliably measure them in a veterinary clinic [2]. Furthermore, the calculations involved in these methods are complex. These limitations may hinder the adoption of such methods in veterinary practice and prevent further research on their clinical reliability and validity. Thus, developing simple, reliable, and stable methods is necessary for assessing body fat in cats.

### 2.3. Simple Evaluation Method for Muscularity

Accumulated fat can mask reductions in muscle mass in aging cats with sarcopenic obesity, resulting in assessments that appear to indicate normal weight. Thus, evaluating muscle mass along with assessing the nutritional status in aging animals is important.

#### Muscle Condition Score

Dual-energy X-ray absorptiometry (DEXA), computed tomography (CT), and magnetic resonance imaging (MRI) are considered gold standards for muscle mass assessment; however, they are impractical in clinical practice owing to limitations similar to those of fat measurements. To address this issue, the WSAVA introduced the Muscle Condition Score (MCS), a simple, globally recognized method [21]. The MCS is a 4-point feline muscle mass scoring system defined as follows: 3, normal muscle mass; 2, slight wasting; 1, moderate wasting; 0, severe wasting [44]. According to Michel et al. [21], the reproducibility and reliability of this method were confirmed in three separate assessments of normal and severely wasted cats. Inter-rater agreement (reproducibility) was moderate (normal: κ = 0.48–0.53; severely wasted: κ = 0.49–0.59). Although intra-rater agreement was practically acceptable (κ = 0.71–0.73), it was not particularly high in terms of accuracy.

Similar to the BCS, the MCS is a subjective method based on visual inspection and palpation. Although pet owners can apply the method themselves, variations in judgment may occur depending on the evaluator’s skill. Additionally, the method is restricted in cats with long hair, senior status, or accumulated fat pads [44]. Similar to body fat assessment methods, a muscle mass assessment method that is easy and clinically stable has yet to be established.

### 2.4. Assessment of Direct Body Composition

#### DEXA, CT, and MRI

DEXA, CT, and MRI are considered gold standards for evaluating body composition. DEXA uses two photon types with different energy levels. It emits radiation into the body and helps estimate the type and quantity of each tissue based on differences in the attenuation of radiation. Speakman et al. [45] reported the use of DEXA in cats. DEXA can accurately evaluate accumulated fat mass, LBM (such as muscle), and bone mineral content with high accuracy. Furthermore, CT and MRI can quantify and distinguish between fat and LBM using two-dimensional cross-sectional images of the trunk.

Bjørnvad et al. [46] performed multiple DEXA scans on the same subjects (five scans in ventral recumbency and two scans in dorsal recumbency) to evaluate measurement variability and accuracy as affected by positioning (ventral and dorsal recumbency). The results showed that in cats, all measurements—total body mass, bone mineral content, bone mineral density, lean soft tissue mass, and body fat—exhibited a coefficient of variation of less than 1%. Furthermore, the results were generally consistent between positions (ventral vs. dorsal), indicating a small positional difference.

However, these methods have limitations, including radiation exposure and the requirement for anesthesia, which involves a certain level of invasiveness. Moreover, the necessary devices are expensive. The price of DEXA equipment generally ranges from $45,000 to $80,000, making it difficult for primary care veterinary hospitals to introduce it solely for body composition assessment. Given these shortcomings, we developed a clinically feasible approach for evaluating animal body composition.

## 3. Development of a New Nutritional Assessment Method

Our research team attempted to establish an objective obesity assessment method based on skeletal zoometry measurements and a body composition assessment method that can be easily and stably applied in clinical veterinary practices. This study includes several figures and tables using published data from Iwazaki et al. (2022) [47] that have not been published in other reports.

## 4. Materials and Methods

Ovariectomy, food intake, body weight, BCS, blood collection, DEXA, ultrasonography, and skeletal zoometry measurements were performed as previously described [47]. All animal procedures were approved by the University of Illinois Institutional Animal Care and Use Committee prior to experimentation (IACUC #17264).

Twenty healthy female domestic shorthair cats [mean age = 9.5 ± 0.1 months; mean body weight = 3.0 ± 0.1 kg; mean BCS = 5.3 ± 0.1] were used in this study.

Cats were housed individually in cages (1.02 × 0.76 × 0.71 m^3^) during two 1-h feeding periods each day (8–9 a.m. and 3–4 p.m.) at the Edward R. Madigan Animal Facility at the University of Illinois at Urbana-Champaign, Urbana, IL, USA. Water was supplied ad libitum. Temperature and humidity were controlled within a 14-h light:10-h dark cycle. Outside the feeding periods, cats were group-housed and able to socialize and exercise outside their cages.

The experiment comprised three phases: a baseline phase (4 weeks), an energy-restricted phase following ovariectomy (12 weeks), and an ad libitum feeding phase (12 weeks). During the baseline period, a moderate-protein, moderate-fiber (MPMF) diet was administered to support normal growth for all cats. After the baseline period, ovariectomy was performed for 16 cats, and a sham operation was performed for 4 cats. The surgical details are provided by Iwazaki et al. [47]. After surgery, the sham-operated cats were fed the MPMF diet. Half of the spayed cats (*n* = 8) were fed the MPFM diet, while the other half (*n* = 8) were fed a high-protein, high-fiber diet. Details of the diets are provided by Iwazaki et al. [47]. During the energy-restricted phase, all cats were fed twice the amount of the diet needed to maintain their body weight at the end of the phase.

The body weight was measured twice weekly, and the BCS (9-point scale [20]) was measured weekly before feeding in the morning.

Zoometry measurements, ultrasonography, and DEXA scans were performed after the baseline phase and 6, 12, 18, and 24 weeks after ovariectomy.

Just prior to scanning, cats were sedated using an intramuscular injection of ketamine HCL, butorphanol tartrate, and dexmedetomidine. Details regarding sedation and recovery are provided by Iwazaki et al. [47]. After sedation, LBM, fat mass, fat percentage, and bone mineral content under ventral recumbency were evaluated using a Hologic model QDR04500 fan beam X-Ray bone densitometer and the accompanying software (Hologic Inc., Waltham, MA, USA) at the University of Illinois Veterinary Teaching Hospital. After completion of the DEXA scans, atipamezole was injected intramuscularly.

Prior to ultrasonography, the back on the left side of the 6–7th and 13th ribs, and the pit of the stomach, were shaved with a commercial hair clipper.

Using a linear transducer at 10 MHz, a cross-sectional image of the 6–7th rib and just caudal to the 13th rib under right lateral recumbency was captured using ultrasound (Exago, Echo Control Medical, Angoulême, France). Using free software (DataPicker Version 1.2, Blue Moon Factory, Tokyo, Japan), the thickness of subcutaneous adipose tissue just caudal to the 13th rib, back fat thickness (BF), and the thickness of the epaxial muscle (EM) on the 6–7th rib and just caudal to the 13th rib were determined in a manner similar to that performed in cats previously [48,49].

In the zoometry measurements, the head and body lengths were determined from the top of the nose to the joint between the sacrum and coccyx. The length from the patella to calcaneus (PCL) was also determined. The circumferences around the 9th and 13th ribs were determined as chest girth (CG) and abdominal girth, respectively. The fBMI was determined by dividing body weight (kg) by the PCL (m) in a manner similar to that performed in cats previously [48,50]. All ultrasound and zoometry measurements were performed by a single investigator (E.I.) trained over several years.

The calculated fat percentage (CFP) (Equation (1)) was determined as follows: {1.54 × CG (cm)} − {1.58 × PCL (cm)} − 8.67 [30]. CFP as Equation (2) was determined as follows: [{CG (cm)/0.7067} − PCL (cm)]/0.9156 − PCL (cm) [29].

### Statistical Analysis

To evaluate the relationship between body composition measured using DEXA and that measured using various zoometry-based methods, linear regression analysis was performed. The fBMI, BF at the 13th rib, CFP1, CFP2, and BCS were each regressed against either FM (g) or fat percentage (%) as the dependent variables. The EMs at the 13th and 6–7th ribs were each regressed against LBM (g) as the dependent value. Moreover, the EMs at the 13th and 6–7th ribs were each regressed against either FM (g) or fat percentage (%) as the dependent values.

Linear regression analyses were performed using R (version 2.8.1). R, R^2^, 95% confidence intervals, and standard errors were calculated. Statistical significance was set at *p* < 0.05.

## 5. Results

The following results are based on the data published by Iwazaki et al. [47].

**Figure 1:**
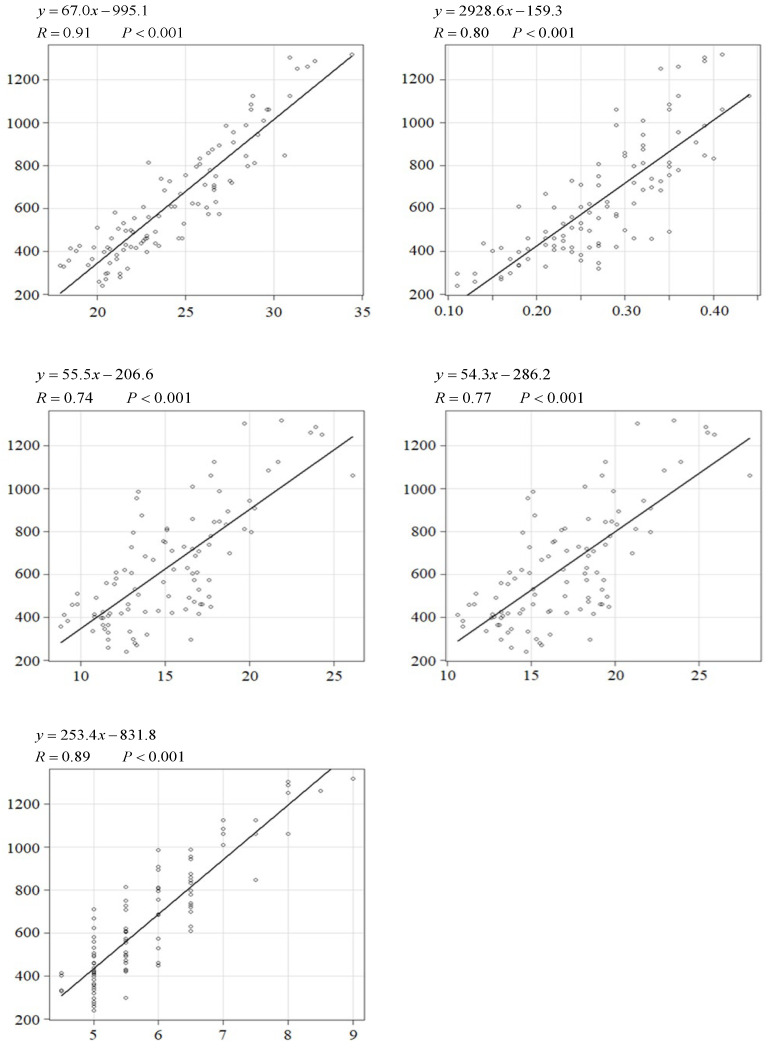
Relationships between fat mass (g) and various parameters in cats. The regression coefficient (R), formula, and *p*-value are presented along with individual data points and the respective regression line. FM (g) was considered the dependent variable. The other parameters were set as independent variables. A regression was considered statistically significant at *p* < 0.05. FM, total fat mass; fBMI, feline body mass index; 13th BF, back fat thickness at the 13th rib; CFP, calculated fat percentage; BCS, body condition score.

Total fat mass was significantly correlated with the fBMI, 13th BF, CFP1, CFP2, and BCS (R = 0.91, 0.80, 0.74, 0.77, and 0.89; R^2^ = 0.83, 0.64, 0.55, 0.51, 0.79; 95% CI = 60.9–73.1, 2494.5–3362.6, 45.5–65.6, 43.9–64.8, 227.3–279.6; SE = 3.1, 218.7, 5.1, 5.3, 5.3, 13.2) (*p* < 0.001) (Figure 1).

**Figure 2:**
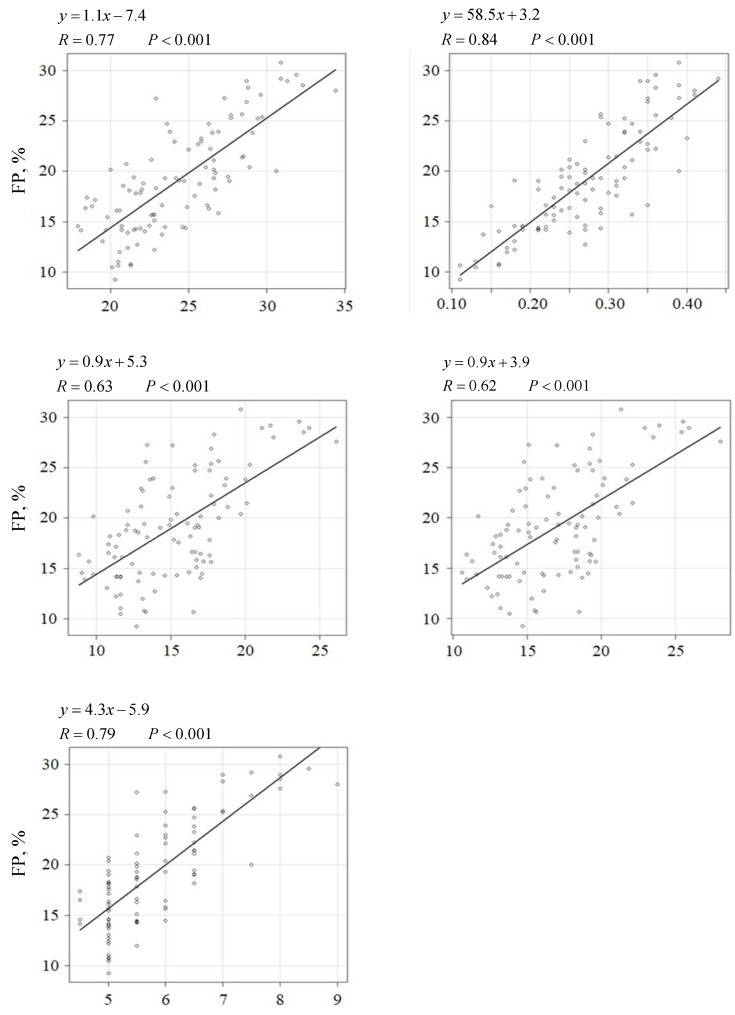
Relationships between fat percentage (%) and various parameters in cats. The regression coefficient (R), formula, and *p*-value are presented along with individual data points and the respective regression line. The fat percentage was set as the dependent variable. The other parameters were set as the independent variables. A regression was considered statistically significant at *p* < 0.05. FP, fat percentage; fBMI, feline body mass index; 13th BF, back fat thickness at the 13th rib; CFP, calculated fat percentage; BCS, body condition score.

The body fat percentage was significantly correlated with the fBMI, BF at the 13th rib, CFP1, CFP2, and BCS (R = 0.77, 0.84, 0.63, 0.62, and 0.79; R^2^ = 0.59, 0.70, 0.39, 0.38, 0.62; 95% CI = 0.9–1.3, 50.8–66.2, 0.7–1.1, 0.7–1.1, 3.7–5.0; SE = 0.1, 3.9, 0.1, 0.1, 0.3) (*p* < 0.001) (Figure 2).

**Figure 3:**
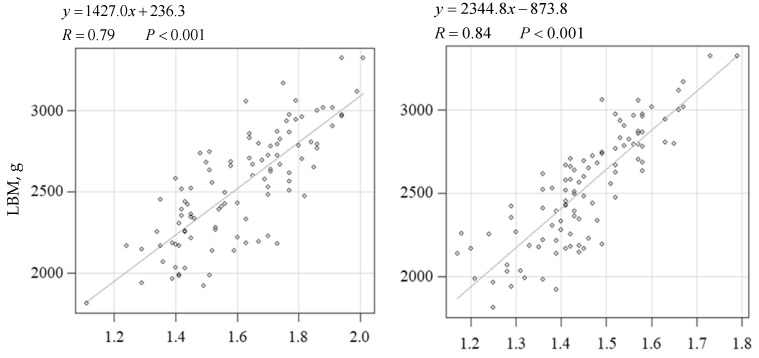
Relationships between lean body mass (g) and various parameters in cats. The regression coefficient (R), formula, and *p*-value are presented along with individual data points and the respective regression line. Lean body mass (g) was set as the dependent variable. The other parameters were set as the independent variables. A regression was considered statistically significant at *p* < 0.05. LBM, lean body mass; 13th EM, epaxial muscle thickness at the 13th rib. 6–7th EM, epaxial muscle thickness at the 6–7th ribs.

LBM was significantly associated with EM at the 13th and 6th–7th ribs (R = 0.79 and 0.84; R^2^ = 0.62 and 0.70; 95% CI = 1203.5–1650.4 and 2041.3–2648.2; SE = 112.6 and 152.9) (*p* < 0.001) (Figure 3).

**Figure 4:**
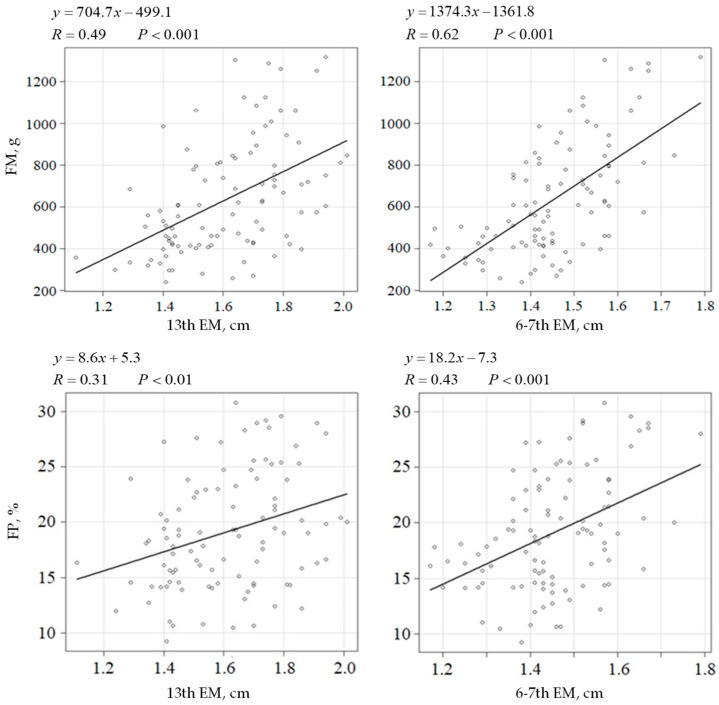
Comparison of fat mass, fat percentage, and epaxial muscle at the 13th or 6–7th rib. The regression coefficient (R), formula, and *p*-value are presented along with individual data points and the respective regression line. Fat mass (g) and fat percentage (%) were the dependent variables. The epaxial muscle thickness at the 13th or 6–7th ribs was set as the independent variable. A regression was considered statistically significant at *p* < 0.05. FM, total fat mass; FP, fat percentage; 13th EM, epaxial muscle thickness at the 13th rib, 6–7th EM, epaxial muscle thickness at the 6–7th ribs.

Total fat mass was significantly correlated with EM at the 13th and 6th–7th ribs (R = 0.49 and 0.62; R^2^ = 0.23 and 0.38; 95% CI = 453.3–956.1 and 1025.5–1723.1; SE = 126.6 and 175.8) (*p* < 0.001) (Figure 4). Additionally, the fat percentage was significantly associated with EM at the 13th and 6th–7th ribs (R = 0.31 and 0.43; R^2^ = 0.09 and 0.17; 95% CI = 3.3–13.8; SE = 2.7 and 3.9) (*p* < 0.01 and *p* < 0.001, respectively) (Figure 4).

The regression formulas (67.0×fBMI (kg/m)−995.1 and 2928.6×13th BF(cm)−159.3) allowed for the estimation of the approximate total fat mass (Figure 1). Additionally, the regression formulas (1.1×fBMI (kg/m)−7.4 and $58.5\;\times \;13^{th}BF\;(cm)+3.2$) facilitated the estimation of the approximate body fat percentage (Figure 1).

The regression formula (1427.0×13thEM(cm)+236.3) facilitated the estimation of the approximate LBM (g; Figure 3).

## 6. Discussion

Adiposity and muscularity are increasingly evaluated for the early diagnosis of several nutritional conditions, such as obesity and sarcopenia, in veterinary practice.

The results of this study demonstrated that the fBMI and ultrasonography for the evaluation of adiposity and muscularity accurately reflected total fat mass, fat percentage, and LBM based on DEXA scans (Figure 1 and Figure 2).

The regression coefficient was higher for the fBMI than for conventional measures, such as BCS, CFP1, and CFP2 (Figure 1), suggesting that the fBMI allows for an accurate evaluation of body fat accumulation.

The fBMI is a comparatively new objective method for assessing nutritional status based on the skeletal length derived from zoometric measurements. Unlike the traditional method for diagnosing obesity―BCS―the fBMI is objective. Moreover, given that it does not include joints in skeletal length [50,51], the fBMI is less influenced by posture and is largely unaffected by fluctuations in body weight, unlike Nelson’s BMI [33] or the FBMI [30,41]. Additionally, it does not require blood sampling or anesthesia and is minimally invasive. Furthermore, it reflects alterations in blood lipids (triglycerides and NEFAs) during the early obesity stages (fBMI = 28.0) before metabolic syndrome onset, enabling precise evaluation of early-stage obesity [50,51].

The temporarily established evaluation criteria for the fBMI in a previous study were updated based on these findings (Table 1) [50,51]. Each level of the total fat mass criterion was calculated using the formula shown in Figure 1. Furthermore, the fat percentage values for cats based on the fBMI ranged between 23.0–27.9 were 13.0–27.9%; we thus set this range as the appropriate total fat percentage range. This is consistent with the BCS criteria [52]. Because the LBM values for cats based on the fBMI range of 23.0–27.9 were approximately 2000–3100, we set this range as the appropriate LBM range.

However, the BF at the 13th rib had a higher correlation coefficient than the conventional BCS, CFP1, CFP2, and fBMI (Figure 2), suggesting that BF at the 13th rib can be used to estimate body fat percentage with high accuracy. Furthermore, EM at the 13th rib was strongly correlated with the LBM (Figure 3), suggesting that it can be used to estimate muscle mass with high accuracy.

Ultrasonography is a practical alternative to DEXA, CT, and MRI. It has been confirmed to be accurate and reliable in bovines, sheep, rabbits, swine, dogs, and humans [53,54,55]. Moreover, the availability and repeatability of ultrasonography for the evaluation of feline adiposity and muscularity were confirmed in our previous study [48,49]. Additionally, we used the three regression formulae, developed based on the relationship between DEXA and ultrasonography results in the current study, for the calculation of the approximate total fat mass, body fat percentage, and LBM. This method enables simple estimation of these parameters in veterinary clinics with higher accuracy than that of CFP1 and CFP2.

Although the EM at the 6–7th ribs was slightly more strongly correlated with LBM than that at the 13th rib, it was more influenced by fat accumulation (Figure 4). This coincides with the differences in the flesh grading systems between the Japan Meat Grading Association and the United States Department of Agriculture [56,57]. The Japan Meat Grading Association assesses the marbling of intramuscular fat using cross-sectional images of the longissimus muscle between the 6th and 7th ribs. Contrastingly, the grading system of the United States Department of Agriculture puts more emphasis on muscularity and maturity, rather than the marbling of intramuscular fat, based on the muscles at the 13th rib. To the best of our knowledge, reports investigating localized fat accumulation accompanying progressive obesity in cats do not exist, and the differences between the meat grading systems in bovine suggests that the EM at the 6–7th rib is more influenced by overfeeding and adiposity than that at the 13th rib. Measurement at the 13th rib enables adiposity and muscularity evaluation in a single procedure, contributing to a decrease in animal and veterinarian stress. Therefore, the 13th rib, rather than the 6–7th ribs, is considered a suitable site for estimating adiposity and muscularity in cats using ultrasonography. The ultrasonographic method can be used in veterinary clinical practice to assess subcutaneous adiposity and longissimus muscularity when DEXA is unavailable [58].

The criteria for the ideal BF at the 13th rib were calculated using the formula shown in Figure 2 by comparing it with the fat percentage (Table 1). The criteria for the ideal EM at the 13th rib were calculated using the formula shown in Figure 3 by comparing them with the LBM.

The maximum total fat mass was 30.8% at the end of the study, suggesting that most female cats exhibited early-stage obesity. Thus, blood lipid levels and hepatic injury markers were within the normal range, and no symptoms of ectopic fat accumulation were observed in the liver. The fat percentage based on the fBMI in this study was relatively lower than that based on BCS and DEXA reported previously [51]. The reason for this discrepancy may be that previous studies recruited cats with a broad age range. This may have resulted in age-related decreases in muscle mass being masked by fat accumulation. In this study, we recruited only healthy young cats, which eliminated the variability associated with age-related changes in fat accumulation and muscle mass. Our results thus provide a high precision assessment of body fat mass and body fat percentage that can be used in veterinary practice during health checks or for setting targets such as weight reduction.

Severe nutritional conditions such as excessive obesity or cachexia can be adequately detected using subjective methods. We thus did not consider these conditions in our study. The fBMI is a valuable tool owing to its sensitivity in detecting early stages of obesity, which typically present with mild metabolic abnormalities. More severe levels of nutritional status, commonly resulting in obvious changes in appearance and metabolism, were not included in this study. Furthermore, we only included female domestic shorthair cats and did not evaluate the effects of sex differences (including males), breed, or sedation status on measurement accuracy. The fBMI may therefore require adjustment for breeds with a short PCL, such as Munchkins; however, related studies have not been reported to date.

Hair length likely does not significantly affect the quality of ultrasonography images because the probe is directly applied to the skin after parting the hair. In fact, clearer ultrasonography images were obtained for long-haired vs. short-haired cats because the hair of long-haired cats was easier to comb through (unpublished data).

Further studies accounting for these factors need to be performed to adapt the method to a broader range of animal statuses.

**Table 1:** Comparison of provisional criteria for the nutritional status in cats.

	BCS	fBMI	13th BF	13th EM	FM	FP	LBM
	(/9)	(kg/m)	(cm)	(cm)	(g)	(%)	(g)
Moderate lean	≤4	≤22.9	≤0.16	≤1.24	≤545.8	≤12.9	≤1999
Ideal	5–6	23.0–27.9	0.17–0.42	1.25–2.00	545.9–880.9	13–27.9	2000–3100
Overweight	7–8	28.0–33.9	0.43≤	-	881.0–1282.8	28.0≤	-
Obese	9≤	34.0≤	-	-	1282.9≤	-	-

BCS, body condition score; fBMI, feline body mass index, 13th BF; back fat thickness on the 13th rib, 13th EM: epaxial muscle thickness at the 13th rib; FM, fat mass; FP, fat percentage; LBM, lean body mass. Criteria for the fBMI were determined similarly to those used in a previous study [55]. Criteria for the 13th BF were determined using FP. Criteria for the 13th EM were determined using LBM. Criteria for the FM were determined using the fBMI. The FP criteria were determined based on the ideal fBMI range of cats in this study. The criteria for LBM were determined based on the ideal fBMI range in this study. This table is based on young female domestic shorthair cats, without including effects of sex differences (including males), breed, or sedation status on measurement accuracy.

## 7. Key Points for Measurement as per the New Nutritional Status Assessment Method

### 7.1. Key Points for fBMI Measurement

The following points describe the PCL measurement required for calculating the fBMI.

**Figure 5:**
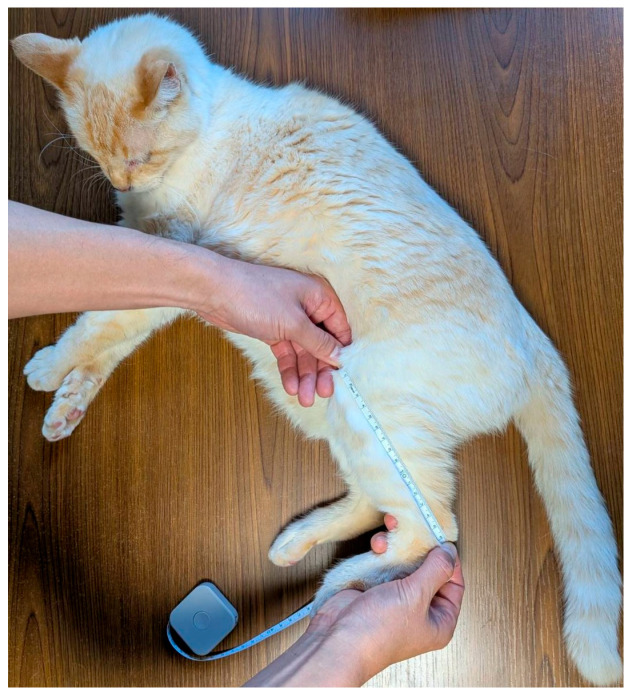
Measurement Position of PCL. The cat was placed in the right lateral recumbent position. The knee was bent at approximately 90°. The distance from the patella to the calcaneus was measured using a caliper or measuring tape.

(1) The cat was placed in the right lateral recumbent position.
(2) To prevent the patella from moving owing to changes in the knee angle, the knee was bent at approximately 90°. At this point, the patella should be located on the tibial extension.
(3) The distance from the patella to the calcaneus was measured using a caliper or measuring tape. An extended caliper was placed against the patella, and the movable part of the caliper was gradually shortened for optimal measurement, given that applying pressure may cause discomfort (Figure 5).

### 7.2. Key Points for Echo Measurement

The following text describes the echo scanning procedure used to measure subcutaneous fat and muscle thickness in cats.

(1) The cat was placed in a right lateral recumbent position.
(2) To the dorsal side of the 13th rib, 70% ethanol was applied, hair in the measurement area was combed, and a horizontal division line parallel to the rib was created.
(3) A linear probe was attached to the ultrasound device and set to 8–10 MHz.
(4) As a couplant, 70% ethanol was used and applied sufficiently to the division line. The probe was positioned on the hair division line so that it was horizontal to the ribs. When performing ultrasonography without shaving, ultrasound gel requires time to penetrate the fur and tends to trap bubbles within. Ethanol was applied because it rapidly penetrates the fur and thus reduces the time required for ultrasonography and decreases stress on the animal.
(5) The position was adjusted such that the longissimus dorsi muscle was fully visible on the screen, after which the adjustment was stopped.
(6) Subcutaneous fat and muscle thicknesses were then measured using the built-in ruler of the ultrasound device or image measurement software.
(7) Subcutaneous fat thickness was measured as the maximum diameter between the dermis and upper fascia of the posterior serratus muscle.
(8) The thickness of the longissimus dorsi muscle was measured as the maximum diameter at the border between the dorsal and ventral sides.

## 8. Conclusions

We developed an objective technique for assessing obesity and body composition in cats in veterinary practice.

The fBMI was highly correlated with fat mass and enabled accurate assessment of early-stage obesity. In addition, significant reliability was observed between BF at the 13th rib and total fat mass and between EM at the 13th rib and LBM.

We further developed calculation formulae to estimate total fat mass, body fat percentage, and LBM based on subcutaneous adiposity and longissimus muscularity. These methods can assist in evaluating body composition in veterinary clinical practice when DEXA is unavailable. However, we did not test severe nutritional conditions such as excessive obesity or cachexia. Furthermore, we only included female domestic shorthair cats and did not evaluate the effects of sex differences (including males), breed, or sedation status on measurement accuracy. Future studies are thus needed to adapt our method to conditions not evaluated in this study.

## 9. Patents

Eiji Iwazaki is an inventor of an awarded patent on this work (Patent No. JP7502798B2 granted on 11 June 2024 related to the measurement of adiposity by ultrasonography, the patent applicant: Nippon Pet Food Co., Ltd., name of inventor: Eiji Iwazaki, status of application: granted) (Patent No. JP6937339B2 granted on 1 September 2021 related to the measurement of muscularity by ultrasonography, the patent applicant: Nippon Pet Food Co., Ltd., name of inventor: Eiji Iwazaki, status of application: granted), (Patent No. JP6696954B2 granted on 27 April 2020 related to the feline body mass index, the patent applicant: Nippon Pet Food Co., Ltd., name of inventor: Eiji Iwazaki, status of application: granted).

## Data Availability

The datasets presented in this article are not readily available because the data are part of an ongoing research project. Requests to access the datasets should be directed to the corresponding author (E.I.).

## Abbreviations

The following abbreviations are used in this manuscript:

BMI	Body Mass Index
BCS	Body Condition Score
CT	Computed Tomography
DEXA	Dual-energy X-ray absorptiometry
FBMI	Feline Body Fat Index
MCS	Muscle Condition Score
NCDs	Non-Communicable Diseases
NEFA	Non-Esterified Fatty Acid
